# How to Manage Beta-Blockade in Older Heart Failure Patients: A Scoping Review

**DOI:** 10.3390/jcm13072119

**Published:** 2024-04-05

**Authors:** Iris Parrini, Fabiana Lucà, Carmelo Massimiliano Rao, Stefano Cacciatore, Carmine Riccio, Massimo Grimaldi, Michele Massimo Gulizia, Fabrizio Oliva, Felicita Andreotti

**Affiliations:** 1 Department of Cardiology, Mauriziano Hospital, Largo Filippo Turati, 62, 10128 Turin, Italy; irisparrini@libero.it; 2 Cardiology Department, Grande Ospedale Metropolitano Bianchi Melacrino Morelli, Via Melacrino 1, 89124 Reggio Calabria, Italy; massimo.rao@libero.it; 3 Department of Geriatrics, Orthopedics, and Rheumatology, Università Cattolica del Sacro Cuore, L.go F. Vito 1, 00168 Rome, Italy; stefanocaccatore@live.it; 4 Cardiovascular Department, Sant’Anna e San Sebastiano Hospital, Via Ferdinando Palasciano, 81100 Caserta, Italy; carmine.riccio@tin.it; 5 Department of Cardiology, General Regional Hospital “F. Miulli”, 70021 Bari, Italy; m.grimaldi@miulli.it; 6 Cardiology Department, Garibaldi Nesima Hospital, 95122 Catania, Italy; michele.gulizia60@gmail.com; 7 “A. De Gasperis” Cardiovascular Department, Division of Cardiology, ASST Grande Ospedale Metropolitano Niguarda, Piazza dell’Ospedale Maggiore 3, 20162 Milan, Italy; fabri.oliva@gmail.com; 8 Cardiovascular and Respiratory Sciences, Università Cattolica del Sacro Cuore, L.go F. Vito 1, 00168 Rome, Italy; felicita.andreotti@unicatt.it

**Keywords:** beta-blockers, heart failure, HFpEF, HFrEF, ventricular dysfunction, clinical trial, guidelines, aged, frailty, multimorbidity

## Abstract

Beta blockers (BBs) play a crucial role in enhancing the quality of life and extending the survival of patients with heart failure and reduced ejection fraction (HFrEF). Initiating the therapy at low doses and gradually titrating the dose upwards is recommended to ensure therapeutic efficacy while mitigating potential adverse effects. Vigilant monitoring for signs of drug intolerance is necessary, with dose adjustments as required. The management of older HF patients requires a case-centered approach, taking into account individual comorbidities, functional status, and frailty. Older adults, however, are often underrepresented in randomized clinical trials, leading to some uncertainty in management strategies as patients with HF in clinical practice are older than those enrolled in trials. The present article performs a scoping review of the past 25 years of published literature on BBs in older HF patients, focusing on age, outcomes, and tolerability. Twelve studies (eight randomized-controlled and four observational) encompassing 26,426 patients were reviewed. The results indicate that BBs represent a viable treatment for older HFrEF patients, offering benefits in symptom management, cardiac function, and overall outcomes. Their role in HF with preserved EF, however, remains uncertain. Further research is warranted to refine treatment strategies and address specific aspects in older adults, including proper dosing, therapeutic adherence, and tolerability.

## 1. Introduction

Heart failure (HF) is a global public health problem and a significant cause of morbidity and mortality in developed countries, affecting about 64.3 million people worldwide [1]. The prevalence of HF increases with age, particularly in patients over 75–80 years. Although improvements in preventative therapies and the management of comorbidities have reduced the incidence of HF, this condition remains the major cause of hospitalization among older adults [1]. In Europe, the prevalence of HF among individuals aged 80 or older ranges from 15% to 20% [1,2,3,4]. However, older adults have long been underrepresented in randomized controlled trials (RCTs) [5,6]. For this reason, treatment efficacy and the optimal management of HF in older patients remains unclear [7,8]. Older HF patients may exhibit age-related conditions, such as frailty, multimorbidity, reduced drug tolerance, and polypharmacy [9,10,11], which may reduce adherence to medical therapies, increase drug–drug interactions, and contribute to worsening HF [11].

According to the latest guidelines, HF is classified into three types based on the left ventricular ejection fraction (LVEF) [9,10,11,12,13,14], namely HF with reduced ejection fraction (HFrEF), i.e., <41%, HF with mildly reduced ejection fraction (HFmrEF), i.e., with between 41% and 49%, and HF with preserved ejection fraction (HFpEF), i.e., ≥50%. Regarding medical therapy, the current guidelines recommend using four “pillars”, regardless of age. The first three pillars are angiotensin-converting enzyme inhibitors (ACE-I)/angiotensin receptor blockers (ARB) or angiotensin receptor–neprilysin inhibitors (ARNI), mineralocorticoid receptor antagonists (MRA), and beta-receptor blocker agents (BBs) [12,13,14]. The EMPEROR [15] and DELIVER [16] trials demonstrated that, compared to placebo, sodium-glucose cotransporter-2 (SGLT2) inhibitors can reduce heart failure HF hospitalizations (HFH) and cardiovascular mortality by 21% and 18%, respectively. These findings support the idea of considering SGLT2 inhibition as the fourth pillar in HF management. However, older HF patients are reported to receive suboptimal therapy compared to their younger counterparts. This discrepancy arises from a reluctance to prescribe due to concerns over adverse events and lower adherence rates among older individuals. As a result, outcomes for these patients tend to be poorer [17]. It should be noted that guidelines address the crucial role of involving a multidisciplinary team in managing HF in older adults, especially considering the complex needs arising from multimorbidity, frailty, cognitive impairment, and polypharmacy. This team should include a specialized HF cardiologist, nurse, geriatrician, dietician, psychologist, physical therapist, and occupational therapist to ensure comprehensive care [13].

BBs have been shown to reduce both death and HFH [18,19,20,21,22]. However, RCTs provide only sparse evidence of their use in older adults. This review addresses the current evidence on the use of BBs in older patients with HF and explores the existing gaps in knowledge on the topic.

## 2. Materials and Methods

### 2.1. Search Strategy

A literature search was conducted in line with the Preferred Reporting Items for Systematic Reviews and Meta-Analyses (PRISMA) guidelines [23]. The search strategy, developed and agreed upon by two authors (I.P. and F.A.) and approved by a third (F.L.), utilized Boolean search terms: “beta blockers” AND “heart failure” OR “diastolic dysfunction” AND “elderly” OR “older”. The literature search was performed on PubMed and EMBASE databases independently by two investigators (I.P. and F.A.), aiming to identify studies on BB therapy in older adults with HF. Titles and abstracts of all articles published in the last 25 years were assessed. Reference lists of the papers obtained through the literature search were screened in order to include a larger number of relevant studies.

### 2.2. Selection Process

Article selection was based on the following inclusion criteria: (a) studies reporting BBs in HF, or diastolic dysfunction, in terms of efficacy, safety, or tolerability; (b) studies including older adults; (c) studies with cohorts of more than 100 patients; and (d) human studies. The exclusion criteria were: (a) non-comparative studies; (b) lack of usable data concerning efficacy, safety, and tolerability of BBs in HF; (c) studies with patient cohorts comprising 100 or fewer individuals; (d) reviews, meta-analyses, commentaries, and letters.

### 2.3. Quality Assessment

The quality of included studies was assessed using Down and Black’s Checklist for Measuring, which evaluates the quality of randomized and non-randomized studies in terms of reporting, external validity, internal validity, and power. Each checklist component is rated using a binary score (0/1) except for three items, rated on a scale from 0 to 2 and 0 to 5, respectively [24]. Two independent researchers (I.P. and F.L.) conducted the ratings. Divergences were resolved by quantification through Cohen’s kappa [25].

### 2.4. Endpoints and Definitions

The primary endpoints evaluated in this review focused on the safety, efficacy, or tolerability of outcomes related to BBs in HFpEF and HFrEF patients. Some studies defined safety as the composite outcome reduction in HFH and deaths during the follow-up. In other studies, efficacy was defined as composite or non-composite, specifically reducing the number of HFH and overall mortality rates. Tolerability refers to the extent to which patients can bear the adverse effects of BBs without significantly impacting their quality of life or leading to treatment discontinuation. A rigid cutoff to define older age was specifically avoided as the definition varied from study to study, ranging from >65 to >70 or 75, or even 80 years.

## 3. Results

**Figure 1:**
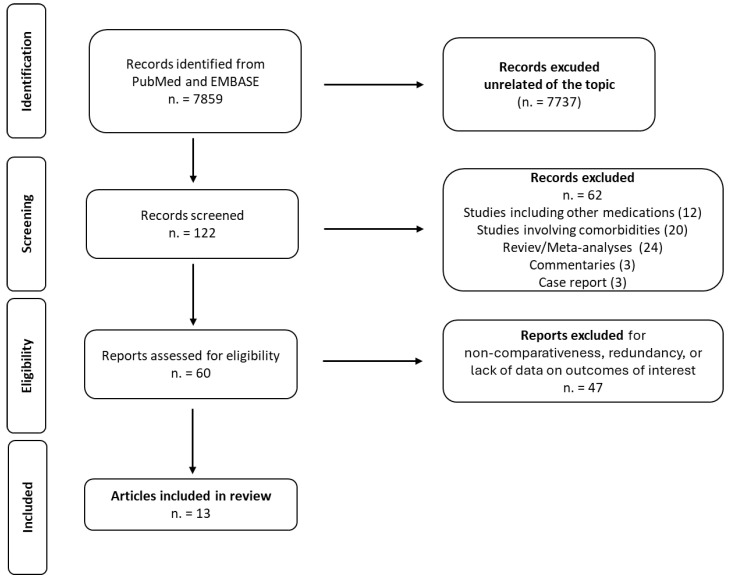
PRISMA flow diagram for literature selection and study process.

The research produced a total of 7859 records. Of these, 7737 were excluded as unrelated to the topic, leaving 122 records to be screened by title and abstract. Following this initial screening, 60 articles were retrieved for further evaluation. Based on our eligibility criteria, 12 articles were selected for inclusion in our review (Figure 1).

A summary of included studies is displayed in Table 1. The studies included in the present review comprise data from a total of 26.426 patients, with either HFrEF [25,26,27,28,29,30], HFpEF [31], or both [20,32,33,34,35].

Eight articles were randomized trials or trial subanalyses [21,26,27,28,29,30,31,32,33,34,35].

Two were prospective observational [29,33], one was cross-sectional [30], and one was a retrospective cohort study [36]. Data from the MERIT-HF trial were reported in two separate articles [27,28]. A single study investigated the effects of nebivolol [21], two investigated bisoprolol [26,33] and one investigated metoprolol controlled-release/extended-release carvedilol [29,31,32,33], and four studies did not specify the type of BB [30,34,35,36].

Follow-up duration ranged from 3 months to 3.3 years.

## 4. Discussion

### 4.1. Heart Failure in Older Adults

The aging population, alongside advancements in the treatment and prognosis of ischemic heart disease and the introduction of effective therapies that enhance survival, are key factors driving an increase in the global prevalence of HF [1,38]. In the USA, nearly the entire population of patients with HF is aged 60 years and older, with about half of those aged 80 years or older [39]. HF in older patients, compared to younger adults, exhibits distinct characteristics from those in younger patients. Older HF patients are predominantly female, with HFpEF being more common. Additionally, they typically carry a high burden of both cardiac and non-cardiac comorbidities, necessitating tailored and multicomponent treatments [39].

**Figure 2:**
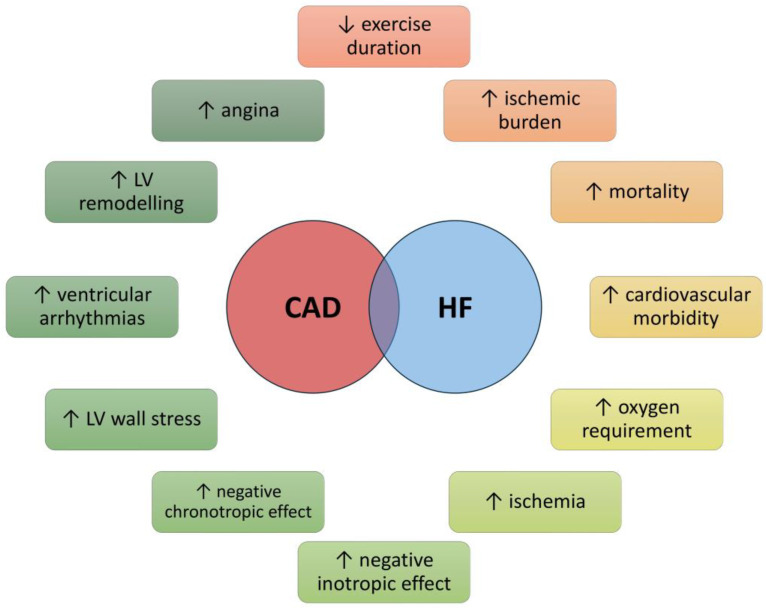
Effects of the coexistence of coronary heart disease and heart failure on patients’ health. Abbreviations: CAD, coronary heart disease; HF, heart failure; LV, left ventricle.

In older individuals, a series of age-related physiological changes significantly contribute to the development of HF. These changes encompass a reduction in myocytes, modifications within the extracellular matrix, enhanced collagen deposition and fibrosis, disturbances in calcium metabolism, and a decline in adenosine triphosphate functionality. Such alterations precipitate compensatory hypertrophy alongside modifications in myocardial contraction and relaxation mechanisms. Moreover, interstitial fibrotic remodeling, matrix degradation, and increased myocardial and vascular stiffness can lead to ventricular dilation, elevated left ventricular (LV) infilling pressures, enlargement of the left atrium, and diastolic dysfunction. The situation is further complicated by comorbid conditions common in this population, including diabetes mellitus (DM), obesity, chronic renal failure (CRF), AH, and AF [40,41]. These comorbidities can potentially activate pro-inflammatory and pro-fibrotic pathways, thereby intensifying cardiac damage through the inflammation of cardiac microvascular endothelial cells and an escalation in oxidative stress [42,43]. Acute or chronic myocardial injury in older patients results in sympathetic activation with chronic adrenergic receptor stimulation, reduced cardiac β-receptor density and responsiveness, and diminished cardiac inotropic reserve. The clinical outcomes of these processes include reduced systolic function, accelerated LV remodeling, and the emergence of life-threatening ventricular arrhythmias [44] (Figure 2).

Older patients have been shown to be less likely to receive an early diagnosis of HF [45]. A study by Oudejans et al. investigated the presence of signs and symptoms of HF in 206 geriatric patients suspected of HF (mean age 82 years). HF was diagnosed in approximately half of the patients, often presenting atypically with symptoms such as loss of appetite and low body mass index. Classic signs and symptoms of HF were absent in one-third of these patients [46].

In older patients, especially women, who were admitted to hospitals for HF, a high prevalence of HFpEF has been reported. The primary clinical manifestations include acute pulmonary edema and arterial hypertension. Older patients with HFpEF were more likely to present with nonspecific symptoms, such as weakness, weight loss, and loss of appetite. Additionally, about 65% of these patients reported respiratory fatigue despite the absence of pulmonary congestion upon physical examination [47,48]. In HFpEF, diastolic dysfunction and LV hypertrophy have been described [47,48]. Blood biomarkers, such as B-type natriuretic peptide (BNP), may not consistently exhibit elevated levels in older HFpEF patients, particularly those in sinus rhythm, with obesity, and/or normal kidney function [49,50]. However, echocardiography and BNP assessment are crucial for making a guideline-directed diagnosis of HF [51,52], and additional biomarkers, such as the soluble circulating form of the suppression of tumorigenicity two receptors (sST2), may guide prognostic stratification on admission [53,54].

In older adults, multimorbidity and polypharmacy, including non-steroidal anti-inflammatory drugs, antidepressants, antiarrhythmics, antibiotics, and anticoagulants, may directly exacerbate HF or amplify the risk of specific drug–drug interactions. Socioeconomic factors, such as limited access to caregivers and specialists, cognitive impairment, and financial constraints, can hinder adherence to medical therapies [55].

Notably, therapeutic options are considered more constrained for older patients due to the pharmacokinetic and pharmacodynamic changes associated with aging. The aging process leads to decreased lean mass and total body water and a relative increase in body fat, contributing to elevated plasma concentrations of hydrophilic drugs and reduced concentrations of lipophilic drugs. Diminished hepatic activity associated with aging disrupts first-pass metabolism, leading to the heightened activation of certain drugs and the diminished activation of others while declining renal function results in decreased clearance of several drugs [56].

### 4.2. Beta Blockers in Older Adults

BBs are a cornerstone in treating HF as they can improve symptoms, reduce hospitalizations, foster LV reverse remodeling, and enhance overall prognosis [57]. However, their utilization in older adults remains limited, largely due to concerns about potential adverse effects such as hypotension, bradycardia, and the risk of exacerbating HF, particularly HFpEF [40].

The previously described pathophysiological changes in the cardiovascular system in older age, coupled with heightened sympathetic nervous system activity, challenge the heart’s ability to function efficiently. BBs play a crucial role in this context by attenuating the adverse effects of sympathetic overdrive, improving myocardial relaxation, and reducing heart rate. This can be particularly beneficial given the altered cardiac physiology in older patients. However, the efficacy and tolerability of BBs in this demographic are influenced by age-related pharmacokinetic and pharmacodynamic variations and the high prevalence of comorbidities [58].

Some concerns have been raised regarding BB use in older patients. By altering the hemodynamic balance, β-blockers transition the patient’s profile from one characterized by normal cardiac output and elevated vascular resistance to one marked by reduced cardiac output and sustained high vascular resistance. This action, along with potential changes to DNA methylation profiles, may age the cardiovascular system of individuals, eventually accelerating the biological aging process [42,59]. Another relevant aspect is the potential role of BBs in increasing the risk of cognitive and functional impairment. According to Steinman et al., the prescription of BBs after acute myocardial infarction in nursing home residents reduced 90-day mortality by 26%. However, they determined an increased risk of functional decline, especially in individuals with significant cognitive or functional impairments [60]. The same study, however, showed that BBs did not negatively affect individuals with normal or mildly impaired cognition and those who maintain independence in activities of daily living [60]. More recently, Holm et al. addressed the association of BBs with an increased risk of developing vascular dementia (hazard ratio 1.72, 95% confidence interval 1.01–3.78; *p* = 0.048), but not all-cause, Alzheimer’s, or mixed dementia [61]. Conversely, data from the Danish national registers suggest that anti-hypertensive treatment with highly blood–brain barrier permeable BBs (e.g., carvedilol) may reduce the risk of Alzheimer’s disease by favoring the elimination of waste brain metabolites [62]. Regarding physical performance, Priel et al. demonstrated that BBs helped minimize exercise intolerance and symptoms by increasing oxygen pulse [63].

BBs are not recommended in HFpEF, which is a multifaceted clinical syndrome affecting various organ systems, accompanied by comorbidities such as AF, AH, DM, and obesity [64]. Conversely, in HFrEF, BBs are advised to manage heart rate, diminish LV hypertrophy, and mitigate ventricular arrhythmias, which are especially detrimental in this patient group [40]. The current review underscores a significant disconnection between clinical research and HF prevalence and demographic trends of incidence. A crucial point raised is the scarcity of recent evidence, which reveals a critical gap in our understanding. This deficiency highlights the urgent need to incorporate older age groups into large-scale international trials, reflecting their substantial presence within the real-world patient demographic. This inclusion is essential to bridge the gap between research and practice, ensuring that findings directly apply to the population most affected by HF, thereby enhancing patient care and outcomes.

### 4.3. Beta Blockers in Older Adults with Predominantly HFrEF

BBs are pivotal for managing patients with HFrEF, significantly enhancing prognosis. Nonetheless, their use among older adults, particularly those over 80, remains sparse. Research exploring BB prescription rates, adherence, tolerability, dose achievement, and their relationship with clinical outcomes has been undertaken, but findings are especially limited for this age group. The CHAMP-HF registry, capturing data from 3518 individuals with HFrEF between 2015 and 2017, reported BB prescriptions at 67% across the cohort, although only 28% received target doses at the 12-month mark. Older age, renal failure, lower blood pressure, and recent HFH were independent predictors of lower BB utilization and dosage [35].

A meta-analysis of 11 RCTs involving 13,833 patients with HFrEF in sinus rhythm, with a median age of 64, noted a decrease in mortality risk across all age groups for those treated with BBs. Drug discontinuation rates were comparable across age groups, with withdrawals primarily due to hypotension, bradycardia, worsening HF, and renal failure. Notably, the benefit of BBs in reducing hospitalization risk diminished with age [65]. The SENIORS trial, the only RCT targeting those aged ≥70, which evaluated BB effects on mortality and hospitalization regardless of LVEF, found a significant reduction in the combined risk of death and cardiovascular rehospitalization as the primary outcome, without a marked decrease in all-cause or cardiovascular mortality alone [21].

A secondary analysis from the TOPCAT trial indicated an association between BB use in patients with HFrEF and an increased risk of readmission for HF, although not for cardiovascular death [34]. Observational data, supported by subanalyses from the MERIT-HF, SENIORS, and MOCHA studies, suggest a reduction in mortality irrespective of BB dosage [26,27,64]. Moreover, an individual patient data meta-analysis of placebo-controlled RCTs by Kotecha et al. revealed an 18% mortality risk reduction for every five beats/min reduction in heart rate (HR) achieved through BB treatment, without a significant link between BB dosing and all-cause mortality [65].

Trials such as CIBIS-II [26], MERIT-HF [27,28], and COPERNICUS [32,66] demonstrated improved survival rates with bisoprolol, metoprolol CR/XL, and carvedilol for patients diagnosed with HFrEF. These studies typically involved younger participants compared to the SENIORS trial [21], with mean ages of 61, 64, and 63 years, respectively, against 76 years in the SENIORS trial [26,27,28,32,66]. Bisoprolol, carvedilol, and metoprolol have thus been endorsed in American and European guidelines as first-line BBs for HFrEF patients [12,13,14]. Nebivolol has also been recommended in European guidelines for HFrEF despite its uncertain effects on cardiovascular or all-cause mortality [12,13].

However, the potential poor tolerability of BBs in older patients may underlie their limited use. The CIBIS-ELD trial, comparing bisoprolol and carvedilol tolerability in individuals over 65 with HFrEF or HFpEF, evaluated target dose achievement (50–100 mg/day for carvedilol and 10 mg/day for bisoprolol) after 12 weeks [33]. Significant proportions, of 19% of patients with HFpEF and 27% with HFrEF, did not reach the target dose, with similar tolerability observed for both drugs. Compared to baseline, patients with HFrEF achieved greater clinical status and LVEF improvements, whereas those with HFpEF experienced more side effects for the same HR reduction [33]. In the CIBIS-II study, 63% of patients achieved the maximum bisoprolol maintenance dose (10 mg/day), and 37% reached 5 or 7.5 mg/day doses [26]. The tolerability was 85% (15% discontinuation rate) [26]. The percentage of patients who reached the target was not higher than in CIBIS-ELD (73%) [33], despite the ten-year-older average age in the latter compared to CIBIS-II patients [26]. The same consideration concerning CIBIS-ELD [33] can be made in relation to COPERNICUS [32,66], MERIT-HF [27,28], and SENIORS [21]. A meta-analysis of RCTs showed that withdrawal of therapy was not correlated with age [65].

The COLA II study assessed carvedilol tolerability in 1030 individuals older than 70 with HFrEF for 6 months. Approximately 80% tolerated the medication, although the maximum dose was seldom achieved, with mean doses varying by age group. The mean achieved dose was 33.3 mg/day and 29.3 mg/day in age groups 70–75 and >80 years, respectively. Advanced age and lower systolic or diastolic blood pressure, but not lower HR, were associated with lower tolerability [31]. A propensity score-matched analysis of the Swedish Heart Failure Registry highlighted that for individuals over 80, ACE-I and BB therapy correlated with enhanced survival without a rise in syncope-related hospitalizations [30]. Resting HR increases were linked to higher mortality in older outpatients with HFrEF [30], emphasizing HR achievement over BB dosing for survival benefits [37]. The STRONG-HF trial underscored that intensive guideline-directed medical therapy titration within two weeks post-discharge, with twice-monthly visits up to 2 months, could significantly enhance patient outcomes compared to usual care [67]. During hospitalization, the target BB dose was met at 55%. An intensive treatment strategy reduced symptoms, improved quality of life, and reduced the risk of 180-day all-cause death or HFH compared to usual care [67].

### 4.4. Beta Blockers in Older Adults with Predominantly HFpEF

In contrast to the well-established benefits of BBs therapy for patients with HFrEF, the evidence supporting their use in HFpEF is less conclusive, mainly due to the lack of specific data from RCTs. The TOPCAT trial, which included patients with preserved or mildly reduced LVEF, where around 80% were receiving BB treatment, highlights this evidence gap [34]. TOPCAT secondary analysis reported that BB therapy was associated with a higher risk of HHF in patients with preserved EF than those with HFmrEF [34]. An increase in resting HR compensates for decreased cardiac output, but significant elevations lead to increased oxygen consumption and coronary distress, predicting mortality, particularly in older patients. Patel et al. reported a 17% higher risk of HFH among HFpEF participants in the OPTIMIZE-HF registry [68]. Furthermore, the J-DHF study suggested that carvedilol might lower the rate of adverse clinical outcomes, notably in patients with advanced diastolic dysfunction [29].

Compared to conventional therapy, Takeda et al. demonstrated that carvedilol alleviated HF symptoms and neurohormonal activation in patients with HFpEF [69]. Data from three European and American registries—the EURObservational Research Program Long-Term Heart Failure (EORP LT-HF) [70,71], OPTIMIZE [36], and Get With The Guidelines (GWTG) [72]—indicate that older HF patients exhibit higher mortality, morbidity, and rates of HFH than younger patients. Conversely, other studies have indicated that HFpEF patients often experience chronotropic incompetence, with the addition of BBs potentially worsening the ability to increase HR and impair exercise capacity [73,74].

Notably, evidence addressing the use of BBs in HFpEF is predominantly derived from studies conducted in Japan. This concentration of studies in Japan may be attributed to the rising prevalence of HFpEF within the Japanese population, a trend closely linked to the aging demographic [75]. However, the applicability of findings from Japanese studies on BB therapy in HFpEF to other ethnic groups may be impacted by several factors, including genetic differences, variations in population health profiles, lifestyle and environmental factors, and the different prevalence of comorbid conditions. As observed by Obokata et al., Japanese people living with HFpEF show relevant phenotypic differences from Western HFpEF patients, including lower rates of obesity and higher rates of DM, AF, and CRF [76]. These considerations highlight the necessity for diverse, multinational research efforts to ensure the global relevance of treatment guidelines for HFpEF.

### 4.5. Adherence to Beta-Blocking Therapy and Multidisciplinary Approach in HF

Compared to other guideline-directed medical therapies, BBs, particularly at target doses, have significantly improved clinical outcomes in younger patients [77]. However, in older adults, the benefits of BBs are not as pronounced, which could be attributed to the low statistical power from arising from the relatively small subgroup analyses. Additionally, the challenges in both administering treatment and achieving target doses in older adults may be due to the higher prevalence of multimorbidity and frailty within this group. Historically, BBs have been utilized to address comorbidities associated with HFpEF, such as arterial hypertension (AH), coronary artery disease (CAD), and atrial fibrillation (AF). However, no evidence suggests BBs confer additional benefits for HFpEF patients without these comorbidities.

**Figure 3:**
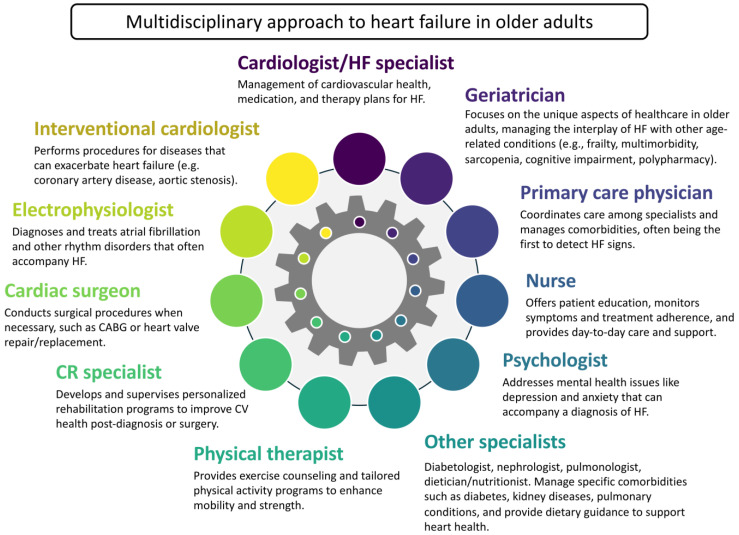
A multidisciplinary approach to HF in older adults. Abbreviations: CABG, coronary artery bypass graft surgery; CR, cardiac rehabilitation; CV, cardiovascular; HF, heart failure.

Adhering to medical therapy is crucial for managing multiple comorbidities in older patients, potentially improving outcomes. The use of cardio-selective BBs in a once-daily dosage and their gradual titration can enhance adherence and minimize adverse effects, particularly in patients prone to hypotension due to polypharmacy with diuretics, antihypertensives, and psychotropic medications. The role of a multidisciplinary team, including physicians, patients, and caregivers, in providing education and engagement with patients and their families is vital for successful management [78]. Collaboration between cardiologists and specialists across various disciplines is essential for delivering comprehensive, integrated care and follow-up (Figure 3). Deprescribing unnecessary medications to streamline regimens to only those essential should be performed in older adults [79,80]. Beyond pharmacotherapy, the recommendation of aerobic exercises, exercise training, and diet-induced weight loss are also beneficial for this patient group [81].

## 5. Conclusions

**Figure 4:**
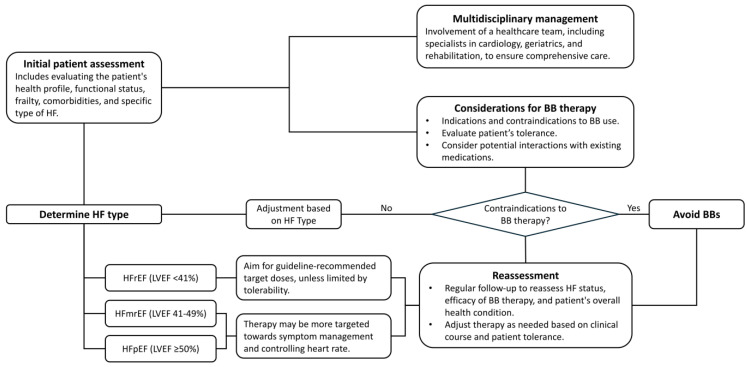
Proposed algorithm for prescribing beta-blockers in older adults with heart failure. Abbreviations: BB: beta-blocker; HF: heart failure; HFmrEF: heart failure with mildly reduced ejection fraction; HfpEF: heart failure with preserved ejection fraction; HfrEF: heart failure with reduced ejection fraction; LVEF: left ventricular ejection fraction.

RCTs on patients with HFrEF showed that BB therapy can reduce mortality and hospitalization by up to 10–40% at the 1-year follow-up. However, these participants are typically younger and have fewer comorbidities, which is not fully representing the broader HF population encountered in real-world settings. Achieving guideline-recommended target doses in older patients remains contentious, as clinical trials predominantly feature younger patients (mean age: 61–65 years). It raises questions about the clinical relevance of reaching these target doses in individuals aged 80 and above, especially considering the increased risk of hypotension and bradycardia, which could lead to falls and other complications in this age group. A practical approach for those over 80 might involve administering the maximum tolerated dose and/or aiming for a target HR to minimize the risk of drug-related adverse effects (Figure 4).

Additionally, the debate over the underuse of beta blockers in older patients is complicated by the high prevalence of HF with preserved ejection fraction (HFpEF) within this demographic. For HFpEF, the prescription of beta blocker therapy is less evidence-based due to a scarcity of data from randomized controlled trials. Indeed, without specific clinical indications such as arrhythmias, AH, or CAD, the literature does not clearly support the use of beta blockers in patients with HFpEF [82], where they may impair exercise capacity and heighten the risk of adverse events. There is a pressing need for further studies that include a significant number of older patients to address these gaps.

## Figures and Tables

**Table 1:** Summary of studies addressing the use of beta blockers in heart failure included in the review.

Study, Year	Country	Study Design	Sample Characteristics	Mean Follow-Up	Objective Main Endpoint	Main Finding	Comment
CIBIS-II, 1999 [26]	Europe	RCT	*n* = 2647 Mean age = 61 Female = 19% HFrEF	1.3 years	Bisoprolol vs. placebo. Mortality.	Bisoprolol reduced both all-cause mortality (11.8 vs. 17.3%, *p* < 0.0001) and sudden deaths (3.6 vs. 6.3%, *p* = 0.0011).	BB therapy was beneficial for stable HF patients. However, safety and efficacy not established in patients with severe NYHA IV class and recent instability.
MERIT-HF, 1999 [28], 2000 [27]	Europe and USA	RCT	*n* = 3991 Mean age = 64 Female = 29% HFrEF	1 year	Metoprolol CR/XL vs. placebo. Mortality.	Metoprolol CR/XL reduced mortality (7.2 vs. 11.0%, *p* = 0.0062) and mortality/hospitalizations (32 vs. 38%, *p* < 0.001).	Metoprolol CR/XL improved survival, reduced HFH, improved NYHA functional class, and had positive effects on patient well-being.
COPERNICUS, 2004 [32]	Europe, North America, Australia	RCT	*n* = 2289 Mean age = 63 Female = 20% HFrEF	10.4 months	Carvedilol vs. placebo. Death and death/hospitalizations by pretreatment SBP strata.	Carvedilol reduced the risk of death and of death/all-cause hospitalizations across BP strata.	The relative magnitude of benefits of carvedilol did not vary by pretreatment SBP. Patients with lower SBP reported higher rates of adverse events.
SENIORS, 2005 [21]	Europe and Brazil	RCT	*n* = 2128 Mean age = 76 (all ≥ 70) Female = 37% HFrEF and HFpEF	21 months	Nebivolol vs. placebo. Death or HFH	Nebivolol reduced death or HFH (31.1 vs. 35.3%, *p* = 0.039).	Nebivolol was well-tolerated in older HF adults.
COLA-II, 2006 [31]	Europe, Asia, South America, Australia	Prospective observational study	*n* = 1009 Mean age = 77 (all > 70) Female = 46% HFrEF and HFpEF	6 months	Carvedilol tolerability (≥3 months of therapy and achieving a maintenance dose ≥12.5 mg/day).	Carvedilol was well tolerated in older adults, even in those with low BP or heart rate (≥70% tolerability).	Older patients with low baseline BP were less likely to tolerate carvedilol. Baseline heart rate did not affect tolerability.
OPTIMIZE-HF, 2009 [36]	USA	Retrospective cohort study	*n* = 7154 Mean age = 80 (all ≥ 65) Female = 42% HFrEF and HFpEF	1 year	Association between incidence of BBs and mortality, hospitalization, and mortality/hospitalization among hospitalized HF patients	BBs were associated with a lower risk of death and hospitalization in HFrEF patients. In HFpEF patients, BBs were not associated with mortality and hospitalizations.	The study highlights the importance of an accurate estimation of LVEF in hospitalized patients before BB initiation.
J-DHF, 2013 [29]	Japan	RCT	*n* = 245 Mean age = 72 Female = 42% HFpEF	3.2 years	Carvedilol vs. no carvedilol. CVD death or HFH.	Carvedilol did not significantly reduce event rates in HFpEF patients overall (24 vs. 27%). In those taking >median doses (>7.5 mg/day), the HR for adverse events was 0.54, *p* = 0.0356.	The standard dose of carvedilol (20 mg/day) but not the lower dose (<7.5 mg/day) might be effective in reducing CVD death and hospitalizations.
CIBIS-ELD, 2016 [33]	Germany, Montenegro, Serbia, Slovenia	RCT	*n* = 876 Mean age = 73 (all > 65) Female = 38% HFrEF and HFpEF	3 months	Carvedilol vs. bisoprolol. Tolerability and clinical measures in HFrEF and HFpEF.	Greater NYHA class improvement in HFrEF vs. HFpEF patients (34 vs. 23%, *p* < 0.001).	Similar target-dose tolerability for carvedilol and bisoprolol. Higher rates of dose-escalation delay and side effects in HFpE vs. HFpEF patients.
CHAMP-HF, 2018 [35]	USA	Cross-sectional observational study	*n* = 1516 Mean age = 66 Female = 29% HFrEF	–	Factors associated with use and dose of recommended HFrEF medications.	Prevalence of known HFH and CAD declined with increasing BB dose.	HFrEF patients received target doses of MRA, but considerably lower than recommended doses of BB, ACEI/ARB, or ARNI.
TOPCAT secondary analysis, 2019 [34]	North and South American subgroup	RCT	*n* = 1761 Mean age = 72 Female = 50% HFpEF = 89%	3.3 years	Main trial: spironolactone vs. placebo. Secondary analysis: association between BB use and HFH/CVD mortality, overall, and by LVEF strata.	BBs associated with higher HFH in patients with LVEF ≥ 50% (HR 1.74, *p* < 0.001), but not in those with LVEF 45–49% (HR 0.68, *p* = 0.39). No association between BB and CVD mortality.	For patients with LVEF ≥ 50%, BBs were associated with increased risk of HFH but not of CVD death.
SwedeHF, 2020 [30]	Sweden	Prospective observational study	*n* = 1732 Mean age = 85 (all > 80) Female = 31% HFrEF	1.76 years	Association between BB use, all-cause mortality, and CVD mortality/HHF.	BBs associated with reduction of all-cause death and CVD death.	Limited generalizability given lower frailty and comorbidity burden compared to other real-world cohorts.
STRONG-HF, 2022 [37]	Multinational	RCT	*n* = 1078 Mean age = 63 Female = 39% Hospitalized HF patients not on full recommended medical therapy	6 months	High-intensity vs. usual care. HHF readmission or all-cause death.	High-intensity care led to a significant reduction in repeat HHF or death at 180 days: 15.2 vs. 23.3%, *p* = 0.002. No significant interactions with age or LVEF categories.	At 90 days, full-dose BB in 49 vs. 4% for high-intensity vs. usual care groups.

Abbreviations: ACEI, angiotensin-converting enzyme inhibitor; ARB, angiotensin receptor blocker; ARNI, angiotensin receptor-neprilysin inhibitor; BB, beta blocker; CAD, coronary artery disease; CR/XL, controlled-release/extended-release; CVD, cardiovascular disease; HF, heart failure; HFpEF, heart failure with preserved ejection fraction; HFrEF, heart failure with reduced ejection fraction; HFH, hospitalization for heart failure; LVEF, left ventricular ejection fraction; MRA, mineralocorticoid receptor antagonist; RCT, randomized controlled trial; SBP, systolic blood pressure.

## Data Availability

No new data were created or analyzed in this study. Data sharing is not applicable to this article.
